# Neuromodulator-dependent synaptic tagging and capture retroactively controls neural coding in spiking neural networks

**DOI:** 10.1038/s41598-022-22430-7

**Published:** 2022-10-22

**Authors:** Andrew B. Lehr, Jannik Luboeinski, Christian Tetzlaff

**Affiliations:** 1 Department of Computational Neuroscience, University of Göttingen, Göttingen, Germany; 2 Bernstein Center for Computational Neuroscience, University of Göttingen, Göttingen, Germany; 3 Department of Computational Synaptic Physiology, University of Göttingen, Göttingen, Germany

**Keywords:** Computational neuroscience, Learning and memory, Neural circuits, Reward, Synaptic plasticity, Neuroscience

## Abstract

Events that are important to an individual’s life trigger neuromodulator release in brain areas responsible for cognitive and behavioral function. While it is well known that the presence of neuromodulators such as dopamine and norepinephrine is required for memory consolidation, the impact of neuromodulator concentration is, however, less understood. In a recurrent spiking neural network model featuring neuromodulator-dependent synaptic tagging and capture, we study how synaptic memory consolidation depends on the amount of neuromodulator present in the minutes to hours after learning. We find that the storage of rate-based and spike timing-based information is controlled by the level of neuromodulation. Specifically, we find better recall of temporal information for high levels of neuromodulation, while we find better recall of rate-coded spatial patterns for lower neuromodulation, mediated by the selection of different groups of synapses for consolidation. Hence, our results indicate that in minutes to hours after learning, the level of neuromodulation may alter the process of synaptic consolidation to ultimately control which type of information becomes consolidated in the recurrent neural network.

## Introduction

It is commonly thought that neural activity changes synaptic connections to encode the memory of an experience^1–3^. Recall of this memory is, in turn, considered to depend on the same subset of neurons becoming active again^4–7^. Thus, characterizing neural activity during the initial experience and during recall should reveal the neural code that supports memory. As yet, the defining features of neural activity required for neural computation, in particular memory recall, remain a topic of active investigation. On the one hand, information in the brain may be subject to so-called rate-based coding, meaning that information is conveyed by the number of spikes in a particular time interval. On the other hand, it may be subject to temporal (sometimes called spike-based) coding, meaning that the information is conveyed by the timing of individual spikes^8,9^.

Evidence for either scheme has been found in many experimental studies and the two schemes can be expressed to a different degree depending on task, brain region, and experimental variables^10–14^. For instance in macaque somatosensory cortex, the amplitude of tactile vibrations is encoded in firing rates, while the frequency of vibrations is encoded in precise spike patterns^15^. In rat primary and secondary somatosensory cortices, information about textured surfaces is conveyed by both rate and spike time codes carrying approximately independent, complementary information about the stimulus^16^. In crustacean mechanoreceptors, the occurrence of precise spike-timing and robust rate coding has been shown to be negatively correlated in a neuromodulator-dependent manner, with serotonin favoring higher rates and allatostatin favoring precise spike-timing^17^. Across a number of species, hippocampal and entorhinal cortex neurons, crucial for semantic and episodic memory^18^, exhibit firing patterns indicative of both rate and spike-based coding schemes^19–21^. These coding schemes have been shown to vary independently^11^ and the rate code can remain preserved even if the spike-based code is disrupted^22,23^. Furthermore, hippocampal coding can be task-dependent, as temporally precise activity was observed during a working memory task with a temporal component but was absent in non-memory control tasks^24^. Taken together, in both sensory and memory systems, the extent of rate-based and spike-based coding flexibly depends on brain state and task.

A crucial point is that the nature of a task, and therefore the optimal coding strategy, is generally not known beforehand, certainly not to animals in experiments like the ones described above. Instead, it stands to reason that the coding scheme is determined retroactively, after training, when the importance of an experience becomes known through factors such as reward, punishment, surprise, or novelty, which are typically signaled by neuromodulator release^25–27^. It is known that neuromodulators must be present for successful memory formation^28–30^ as well as for initial memory consolidation (synaptic consolidation), for which there is a time window of up to hours in which neuromodulation has an effect^31–36^. Neuromodulator signals can even retroactively affect a memory trace in the minutes to hours *after* the related event took place^27,37–40^. Thus, the effect of selective neuromodulation on consolidation makes it an ideal candidate for retroactively changing the neural representation of an experience.

A potential mechanism for such retroactive control is neuromodulator-dependent synaptic tagging and capture (STC). STC describes the transfer from the early to the late phase of long-term synaptic plasticity and is strongly suggested to underlie synaptic memory consolidation^41–44^. The original STC hypothesis^45^ states that following sufficient stimulation, a synaptic tag is set, which enables the capture of plasticity-related proteins and thereby leads to the stabilization of synaptic change on a timescale of minutes to hours. Experimental studies show that dopamine and other neuromodulators lower the threshold for the synthesis of these proteins in neurons^46–51^, suggesting a crucial role of neuromodulation not only during learning but also for memory consolidation in the hours after the experience (also cf.^31–34,38,40,41^). Previous theoretical models of STC have, however, only considered a constant protein synthesis threshold without explicitly modeling its dependence on a neuromodulator^43,44,52–54^. Thus, the connection between neuromodulatory influence on late-phase plasticity and memory functionality remains to be explored computationally.

To contribute towards filling this gap, we study memory function at the network level, building on the concept of cell assemblies^1,4,44,55–57^. A cell assembly is formed when a group of neurons receives sufficient stimulation to increase synaptic strengths within the group. Later, if part of the original stimulus is presented to the network, the strong internal connections elicit a spatial firing rate pattern (“spatial” in the sense of network topology) resembling the pattern that was originally present during learning, thereby recalling the memory. This resemblance is thought to support rate coding^4,55,58^. However, the learning stimulus can also lead to outgrowth of the input-defined assembly via the strengthening of synapses connecting to neurons outside the assembly, recruiting them as so-called “support” neurons. The activity patterns of support neurons have been shown to enrich the assembly dynamics and to enhance the learning of temporal sequences^58^.

In this study, we employ the biologically realistic model of memory consolidation from our previous work^44^, which features calcium-based early-phase plasticity and STC, and introduce a neuromodulator dependence for the protein synthesis threshold as suggested by Clopath and colleagues^52^. We show that in our model, the consolidation of synapses connecting to neurons outside an input-defined cell assembly, and thereby the extent of cell assembly outgrowth, critically depends on neuromodulation in the time after learning. Based on this, we show that neuromodulation not only gates whether or not consolidation occurs, but that it also controls the degree to which each type of neural coding is used. The main result of our study is that if the amount of neuromodulator is high enough, cell assembly outgrowth is extensive and the rich dynamics of the support neurons enable information storage by temporal structure. On the other hand, with low amounts of neuromodulator, and hence no outgrowth, only core cell assemblies are formed, featuring improved storing of information in rate-based input-defined structures.

## Results

**Figure 1:**
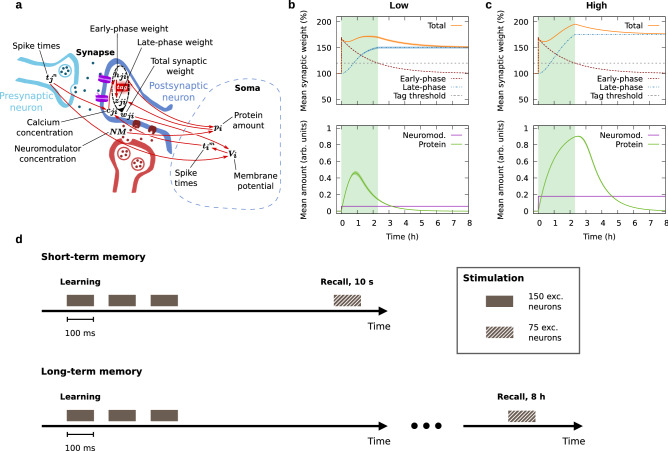
Model and stimulation protocols. (**a**) Synaptic model integrating calcium-based early-phase plasticity and STC-based late-phase plasticity, depending on neuromodulation. (**b**) Mean synaptic weight, neuromodulator, and protein amount within the cell assembly core over time in the case of a low level of neuromodulation ($NM =0.06$). (**c**) Mean synaptic weight, neuromodulator, and protein amount within the cell assembly core over time in the case of a high level of neuromodulation ($NM =0.18$). The green rectangular shading indicates the average lifetime of the synaptic tag. Error bands indicate the standard deviation over 50 trials. (**d**) Stimulation protocol to learn and recall a memory representation. Recall stimulation is applied either 10 s or 8 h after learning, corresponding to short-term and long-term memory, respectively.

The model that we use builds on established experimental and theoretical results^41,44,52,54,59–61^. It features synapses with calcium-based early-phase plasticity and STC-based late-phase plasticity (Fig. 1a). Pre- and postsynaptic spiking activity trigger changes in the postsynaptic calcium concentration, thereby altering the early-phase weight. Sufficient early-phase plasticity at a particular synapse yields the formation of a synaptic tag (Fig. 1b, c). Sufficient early-phase plasticity at many synapses of the same postsynaptic neuron triggers protein synthesis in a neuromodulator-dependent manner. Late-phase plasticity occurs if a synapse is tagged and proteins are available. The total synaptic weight, which determines the magnitude of postsynaptic potentials, is given by the sum of the early- and late-phase weight (cf. “Methods” section).

### Neuromodulation in the hours after learning determines which synapses get consolidated

**Figure 2:**
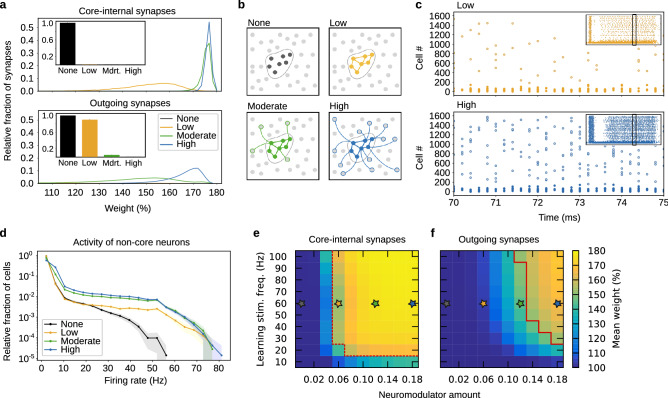
Neuromodulation controls the extent of outgrowth and the activity outside the core assembly. Stars in panels (**e**, **f**) indicate the four different levels of neuromodulation shown in (**a**–**d**). (**a**) Distribution of the weights of core-internal synapses and outgoing synapses at recall after 8 h, averaged via bins of ${\sim }1.64\%$. Insets show the fraction of synapses with no substantial late phase ($<3.26\%$ potentiation, first two bins). (**b**) Sketches of core assembly and outgrowth at four different levels of neuromodulation. Lines represent synapses with consolidated potentiation. (**c**) Spike raster plots showing the spiking of the excitatory neurons during recall stimulation to the first 75 neurons, 8 h after learning. (**d**) Distribution of the firing rates of the excitatory neurons outside the core assembly during recall 8 h after learning. Averaged via bins of 4.16 Hz. (**e**, **f**) Raster plots showing the mean weight of the core-internal synapses and the outgoing synapses 8 h after learning. The dashed and solid red lines demarcate the regimes where the weights are above 150%. All data except the spike raster plots were averaged over 50 trials. Error bands show the 95% confidence interval.

To form a cell assembly in our network, we apply a strong learning stimulus to a subset of 150 excitatory neurons (see Fig. 1d). The stimulus fluctuates around the mean value following an Ornstein-Uhlenbeck process, which is computed individually for each neuron (see “Methods” section). We call the neurons receiving this direct external stimulation during learning the *core assembly*, and the synapses between core assembly neurons *core-internal synapses*. Synapses from the core assembly to the rest of the network are called *outgoing synapses*. If the outgoing synapses are significantly strengthened, we refer to the neurons they connect to as *support neurons* or *outgrowth* (cf. Fig. 2b).

Our first aim was to demonstrate how neuromodulation controls the consolidation of outgoing synaptic weights to enable long-term outgrowth, and to compare this to the impact of the strength of the learning stimulus. To this end, we conducted simulations with various settings of stimulus strength and amount of neuromodulator and analyzed the resulting network 8 h after learning (see Fig. 2e, f). Since we were interested in the effect of neuromodulation on consolidation, neuromodulator levels were held at a constant level for the duration of a simulation ($\sim 8$ h; also see the next subsection for the outcome of other paradigms).

Depending on the amount of neuromodulator, different patterns of consolidation of synaptic weights emerge. As expected, if neuromodulator is not present, then no consolidation of synaptic weights takes place, regardless of the strength of the learning stimulus. Similarly, no consolidation takes place if the frequency of the learning stimulus is too low (Fig. 2a, e, f). With increasing neuromodulator amount and stimulus strength, selected subpopulations of synapses enter the late phase. Core-internal synapses become consolidated even at low levels of neuromodulation after learning (see Fig. 2a, e). Higher levels of neuromodulation, however, lead to consolidation of both core-internal synapses as well as outgoing synapses (see Fig. 2a, f; Supplementary Fig. S7a, b). The distribution of the incoming synapses entering the core assembly, as well as the control synapses outside the core assembly, shows that most of these synapses remain unpotentiated (Supplementary Fig. S1a).

Next, we applied a recall stimulus to half of the neurons that had received the learning stimulus (Fig. 1d). Measuring the spiking activity outside of the core assembly during recall after 8 h, we found that it increases drastically with the amount of neuromodulator that was present during consolidation (Fig. 2c, d), but approaches saturation at moderate to high amounts. The distribution of activity inside the core assembly is shown in Supplementary Fig. S1b.

Considering the amount of protein synthesis that has taken place in the network by 1 h and 2 h after learning (Supplementary Fig. S2), there are much lower protein levels in non-core postsynaptic neurons around 2 h after learning as compared to 1 h after learning. Thus, most of the consolidation of outgoing synapses has to happen early. In contrast, the amount of protein in core neurons increases even further beyond the first hour after learning (“After 2 h”, Supplementary Fig. S2e), indicating that here, STC exerts more impact at later times (also cf. the results on the timing of neuromodulation in Fig. 4). Independent of proteins, consolidation finally stops in all parts of the network around 3 h after learning, by which time all synaptic tags have vanished (cf. the threshold crossing in Fig. 1b, c).

Taken together, neuromodulation gates both whether consolidation via STC takes place and which type of synapses undergo consolidation. While low neuromodulator amount facilitates consolidation of core-internal synapses, higher amounts of neuromodulator promote the late phase of synapses within the core assembly and from the core assembly to neurons throughout the rest of the network. The crucial question that we will address in the next subsection is: Are there functional consequences of these different patterns of neuromodulator-dependent late-phase synaptic weights?

### Neuromodulator-dependent late phase has differential effects on recall of input-defined and self-organized firing rate patterns

**Figure 3:**
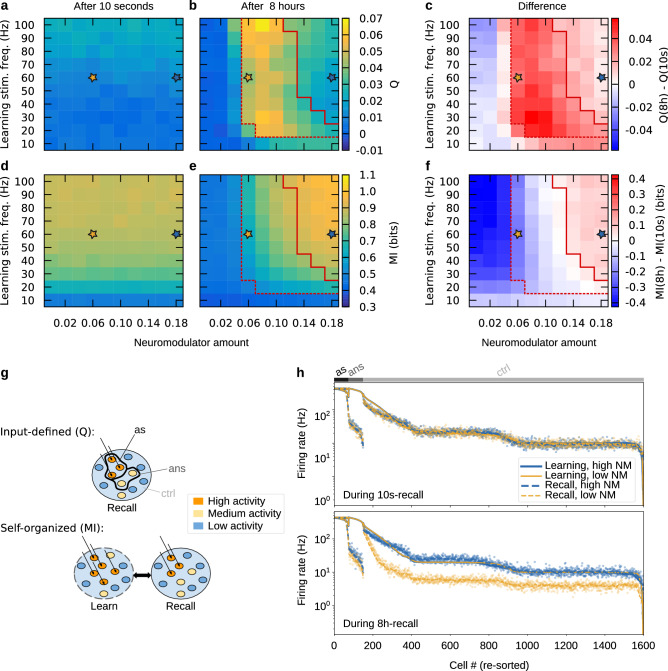
Neuromodulator-dependent consolidation has differential effects on completion of input-defined and self-organized patterns. The performance on completion of an input-defined pattern (**a**) 10 s, and (**b**) 8 h after learning, as measured by the coefficient *Q*, demonstrates the impact (**c**) of neuromodulator-dependent STC. Analogously, panels (**d**–**f**) show the performance on completion of a self-organized pattern, measured by mutual information $MI$. Dashed and solid red lines demarcate where the late-phase weight of core-internal and outgoing synapses reached $150\%$ potentiation, respectively (cf. Fig. 2e, f). (**g**) Depicts the definitions of the two performance measures—*Q* depends on the activities during recall in the subpopulations “as”, “ans”, and “ctrl”, which are defined by the inputs for learning and recall, while the mutual information $MI$ depends on the relationship between the activities during learning and during recall. Colors represent neuronal firing rates. (**h**) Shows the firing rates of the excitatory neurons grouped by subpopulation. Each subpopulation has been sorted according to their firing rate during learning. Data in (**a**–**f**) were averaged over 50 networks.

To investigate the functional consequences of the neuromodulator dependence of late-phase synaptic plasticity, we started by considering the firing rates in our network upon recall stimulation. We used two measures derived from the distribution of firing rates, describing the performance on different pattern completion tasks (cf.^44^): the completion of an input-defined pattern and the completion of a self-organized pattern (Fig. 3g). While the former targets the increased activity in the neuronal subpopulation that has received learning stimulation, the latter targets the altered activity in the whole population of neurons. For these two tasks, we compared the performance directly after learning with the performance 8 h later, that means, after consolidation. During learning we administered stimulation to 150 neurons in three pulses of 100 ms each, whereas to recall, only 75 of these neurons received one 100 ms pulse (cf. Fig. 1d). We measured the completion of an input-defined pattern by the extent (*Q*) to which recall stimulation activated the 75 non-stimulated neurons that had previously received the learning stimulus, compared to the activity in control neurons. On the other hand, for self-organized patterns, we measured the success of pattern completion by the resemblance (mutual information $MI$) of the distribution of firing rates in the network during recall and the distribution during learning.

As expected, we found that 10 s after learning, only the strength of the learning stimulation had an influence on pattern completion (Fig. 3a, d). However, we found that consolidation through STC led to different results 8 h after learning (Fig. 3b, e; Supplementary Fig. S7c, d). Scrutinizing the difference between the two recall times, we found that there are regimes of deterioration and improvement, depending on learning stimulation and neuromodulation (Fig. 3c, f). Remarkably, the completion of an input-defined pattern functions best in an intermediate regime, while it deteriorates at higher neuromodulator concentrations. The completion of a self-organized pattern, however, behaves almost contrary—it exhibits moderate performance for intermediate amounts of neuromodulator but strongly benefits from higher neuromodulator concentrations. The dashed and solid red lines emphasize that these findings are correlated with the outgrowth shown in Fig. 2e, f. The difference in the two measures at the level of firing rates becomes evident from Fig. 3h, which shows that after consolidation, a high neuromodulator amount gives rise to much more activity in the control population, whose neurons are recruited into the self-organized pattern. Note that in the next subsection, we will examine how these rich dynamics can serve to store temporal structures.

**Figure 4:**
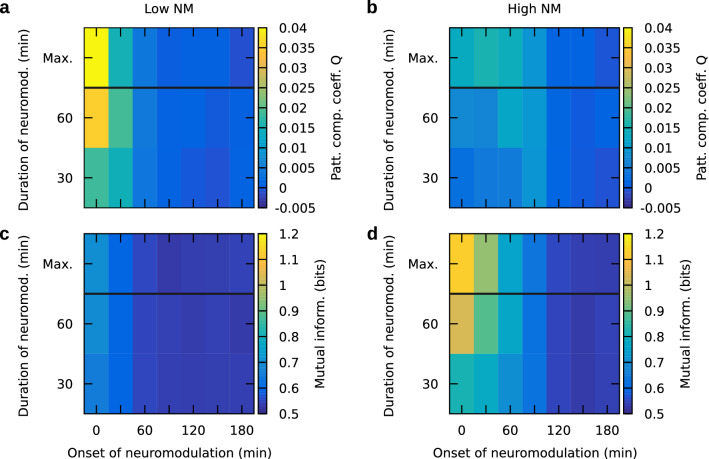
Timing of neuromodulation during consolidation has differential impact on recall of input-defined and self-organized patterns 8 h after learning. (**a**) Low neuromodulation (NM) causes strong recall of an input-defined pattern (measured by *Q*) if starting early and lasting long. (**b**) High neuromodulation causes significant recall of an input-defined pattern (measured by *Q*) only in a certain regime (also cf. Fig. 3b). (**c**, **d**) For self-organized patterns measured by mutual information, both low and high neuromodulation cause better recall if starting early and lasting long. Data were averaged over 50 networks. Frequency of the learning stimulation was 60 Hz. The duration “Max.” indicates the case where neuromodulation is present throughout the whole simulation, otherwise the duration is given in units of minutes.

While we considered neuromodulation throughout the whole simulation for the main part of this study, we also looked into the influence that the timing (i.e., the beginning and end) of neuromodulation could have on memory consolidation. To this end, we considered cases where we restricted neuromodulation to a duration of 30 or $60\,{\text {min}}$. Furthermore, we varied the onset time of neuromodulation between 0 and $180\,{\text {min}}$ after learning. This enabled us to identify the following effects of the timing: (1) low neuromodulation, if starting early and lasting long, causes strong recall of an input-defined pattern (Fig. 4a; Supplementary Fig. S8a) and slightly enhanced recall of a self-organized pattern (Fig. 4c; Supplementary Fig. S8c) and (2) high neuromodulation enables (weak) recall of an input-defined pattern only in a certain regime (Fig. 4b; Supplementary Fig. S8b), while it can cause strong recall of a self-organized pattern if starting early and lasting long (Fig. 4d; Supplementary Fig. S8d). These results are in accordance with our findings on the recall of input-defined and self-organized patterns as a function of neuromodulation and learning stimulation (Fig. 3), and they demonstrate that our model may capture findings of experiments that involve neuromodulator release on timescales of minutes to hours, especially, pertinent findings of behavioral tagging experiments^38^. Furthermore, the simulation results provided here complement a thought experiment on the impact of STC that we will provide in the discussion section of this article.

### High neuromodulator amount promotes outgrowth supporting temporal coding

Next we asked how temporal information in neural activity patterns depends on the presence of neuromodulator during the transfer of synaptic weights to the late phase. We hypothesized that consolidation of temporal patterns would improve with increased levels of neuromodulator for several reasons. First, as we showed in the previous subsection, low neuromodulator levels facilitate consolidation of an input-defined cell assembly, with the remaining neurons firing at low rates (Fig. 3h). With increased neuromodulator levels, however, consolidation of outgoing synapses was improved and neurons outside of the input region were recruited, leading to improved consolidation of a self-organized rate code. The self-organized code consisted of higher and more diverse firing rates outside the input region (Fig. 3h), which we hypothesized could support an increase in temporal information. Second, recruitment of neurons that did not directly receive input stimulation has previously been associated with increased performance on a motor sequence task^58^. If activity patterns from the network are to be used to reliably control a sequence of, for example, motor outputs, then the temporal progression of the activity is important. This suggests recruitment of support neurons via the consolidation of synapses from the assembly to the rest of the network should increase, and possibly stabilize, temporal structure in the activity patterns.

**Figure 5:**
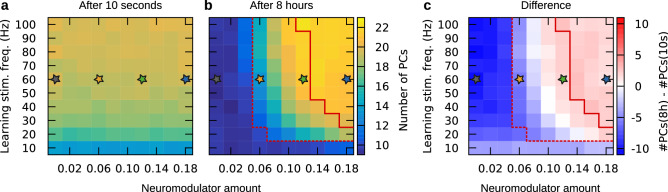
Moderate to high level of neuromodulation during consolidation is required to increase or maintain dimensionality of neuronal activity upon 8 h recall. Number of principal components (PCs) required to explain 70% of variance in 1 ms-binned spike data during (**a**) 10 s recall and (**b**) 8 h recall. (**c**) The difference between 10 s recall and 8 h recall. Data were averaged over 50 networks (**a**, **b**) before being subtracted (**c**). For statistical analysis see main text.

To test whether temporal structure was increased after consolidation specifically for higher neuromodulator concentrations, we first estimated the dimensionality of the neural activity using principal component analysis (PCA). PCA was computed on the binned spike data and the number of principal components required to explain $70\%$ of the variance was counted (see “Methods” section and Supplementary Fig. S3). This was done for three different bin sizes (1 ms, 2 ms, and 4 ms) and the patterns of results were assessed. As expected, during recall at 10 s, dimensionality of the spike data increased with stimulation strength, and neuromodulator concentration had no effect (see Fig. 5). However, during the 8 h recall and thus after neuromodulator-dependent consolidation, temporal structure in the spiking dynamics, as indicated by the dimensionality of the neuronal activity, survived the transfer to the late phase for moderate to high levels of neuromodulator but not for lower neuromodulator concentrations (see Fig. 5 and Supplementary Fig. S4, for statistics see Supplementary Tables S1–S3). In particular, moderate neuromodulation consistently increased dimensionality at 8 h recall compared to 10 s recall for all bin sizes (green star in Fig. 5 and Supplementary Fig. S4). Thus, we observed the highest dimensionality between the best regimes for recall of input-defined and self-organized patterns (cf. previous subsection). Depending on bin size, high neuromodulator levels increased or maintained dimensionality (blue star in Fig. 5 and Supplementary Fig. S4) and low neuromodulator levels reduced or maintained dimensionality at 8 h recall (yellow star in Fig. 5 and Supplementary Fig. S4). The dimensionality of neural activity at 8 h recall for moderate and high neuromodulation were consistently higher than for low neuromodulation (all $p \le 0.001$, Mann-Whitney U tests, two-tailed, see Supplementary Table S4). As expected, when no neuromodulator was present, dimensionality consistently decreased at 8 h recall compared to 10 s recall (gray star in Fig. 5 and Supplementary Fig. S4). Thus we hypothesized that for moderate to high neuromodulator levels, the dimensionality of neural activity would be high enough to support a spike time code. Since, however, high dimensionality does not necessarily translate to stable temporal coding, next we measured spike time stability between learning and recall explicitly.

**Figure 6:**
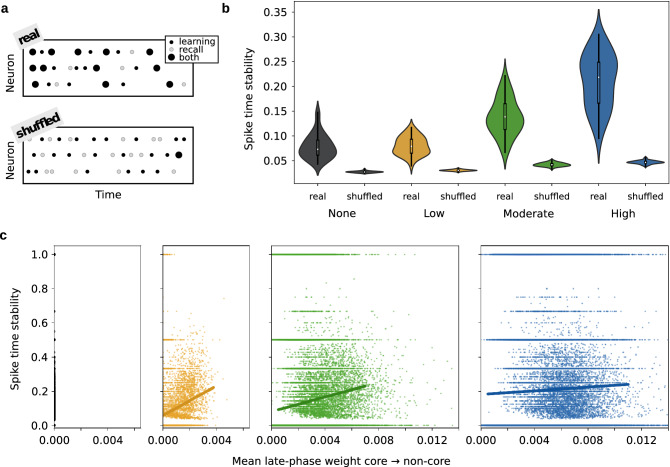
High level of neuromodulation during consolidation promotes spike time codes, particularly in support neurons. (**a**) Schematic of how spike time stability was quantified for real spike data versus shuffled data. Black points indicate the spikes during learning, light gray points the spikes during recall. Stable spike occurrence at the same time is indicated by large black circles. (**b**) Stability of spike times for the final learning stimulus pulse versus 8 h recall as a function of neuromodulator concentration for both real and shuffled data; learning stimulation frequency 60 Hz (i.e., gray, yellow, green, and blue stars from previous figures). Each violin plot contains an inset box-and-whisker plot showing median (white point), quartiles (black bar), and min to max (black line) across 50 network simulations. Kernel density estimates extend vertically beyond the extrema of the data and are normalized horizontally to have the same width. (**c**) Stability of spike times for non-core neurons as a function of mean late-phase weight from the core assembly, separated into none, low, moderate, and high neuromodulator concentration. Colors same as in **(b)**. Each point cloud contains data from 1450 neurons of 50 network simulations. Each point represents the mean late-phase weight of outgoing synapses from the core neurons to a specific non-core neuron.

For the increased temporal structure to be of actual use, temporal information induced by the input during learning should be available during recall. In particular, to achieve a stable spike time code, there should be a subset of neurons in the network with a similar pattern of spike times during learning and recall. To test this, for each neuron we binned the spike times to 1 ms and computed the number of spikes that occurred at the same time during the final pulse of the learning stimulus and during 8 h recall, normalized by the total spikes that the neuron fired during the final pulse of the learning stimulus. For each of the 50 network initializations, we computed a spike time stability score by averaging over all neurons in the network. As a control, for each of the 50 network simulations we shuffled the spike times such that the average firing rate for each neuron remained the same, but the temporal structure was destroyed (Fig. 6a; see “Methods” section). A two-way ANOVA was performed to analyze the effect of neuromodulation and real/shuffled data on spike time stability (Fig. 6b). There was a significant interaction (F[3, 392] = 107.1, $p < 0.0001$), reflecting that differences between real and shuffled data increased with neuromodulator level (see Fig. 6b). The main effects for both neuromodulator level and real/shuffled data were significant (both $p < 0.0001$). Post-hoc two-tailed independent samples t-tests confirmed that for none, low, moderate, and high neuromodulator levels, spike times were significantly more stable in the real data than in the shuffled data (all $p < 0.0001$) and for real data, higher neuromodulator levels led to a more stable spike time code (low vs. moderate, $p < 0.0001$; moderate vs. high, $p < 0.0001$). Spike time stability was, however, not increased by low neuromodulator concentration during consolidation (none vs. low, $p = 0.79$). This indicates that there can indeed be information in the neurons’ spike times and that this temporal code is most stable for higher neuromodulator levels.

We then asked the question whether spike time stability for individual neurons might be linked to the strength of outgrowth after consolidation. In particular, we were interested in whether neurons outside the core assembly receiving strong synapses from the assembly, that means support neurons, had more stable spike time codes (Fig. 6c). For this, we computed the average late phase synaptic weight from the 150 core neurons to each of the other 1450 excitatory neurons in the network. We also computed the spike time stability for each individual neuron (see “Methods” section). This was done for 50 network simulations for none, low, moderate, and high neuromodulator levels, and we found that indeed, spike time stability and average synaptic strength received from the core were correlated (low: $r = 0.29$, $p < 0.0001$, $N = 71{,}471$; moderate: $r = 0.25$, $p < 0.0001$, $N = 71{,}149$; high: $r = 0.13$, $p < 0.0001$, $N = 70{,}705$; Spearman’s rank correlation). Notably, the effect decreased with neuromodulation strength.

Taken together, temporal structure as indicated by PCA dimensionality survived the transfer to the late phase for moderate to high neuromodulator concentrations during consolidation. Interestingly, dimensionality was most increased by consolidation under moderate neuromodulator levels. However, high dimensionality was not a sufficient condition for high spike time stability. Spike time codes were more stable for higher neuromodulator levels during consolidation. And the stability of a individual neuron’s spike time code increased with the strength of synaptic connections it receives from the core assembly, indicating a critical role for support neurons in stable temporal coding. In the next subsection, we will investigate whether the dimensionality and spike time stability under higher levels of neuromodulation are sufficient to store and “read out” temporal sequences from the network activity.

### Neuromodulator-dependent consolidation of outgoing synapses improves storage of temporal sequences

**Figure 7:**
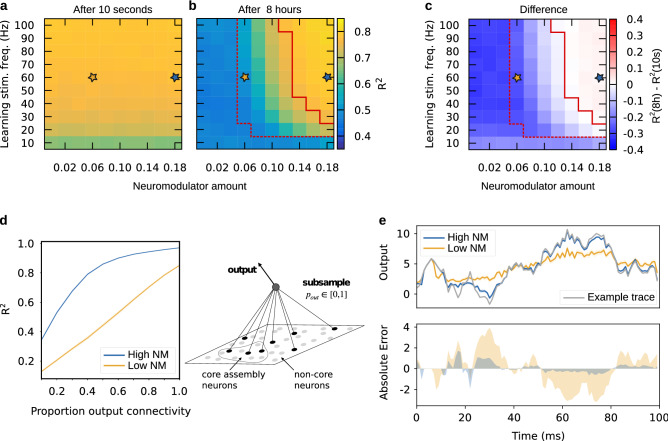
High level of neuromodulation during consolidation supports readout of temporal sequences. The goodness of fit ($R^2$) of the regularized linear regression model is shown for recall (**a**) 10 s and (**b**) 8 h after learning. Yellow star represents low neuromodulator condition, blue star high neuromodulator. (**c**) The impact of neuromodulator-dependent STC on the shared temporal information during learning and recall is measured as the difference between the 8 h and 10 s results. (**d**) The goodness of fit is shown for high (blue) and low (yellow) across different percent output connectivities. A depiction of the subsampling process is shown on the right. Here we sample from all 1600 excitatory neurons, see Supplementary Figs. S5 and S6 for sampling from *only* core and *only* non-core neurons. (**e**) The predicted trajectories at 8 h recall in the low (yellow) and high (blue) neuromodulator conditions are shown for one example target function (gray). The bottom panel shows the absolute error (with sign). Data in (**a**–**c**) were averaged over 5000 instances: 50 networks with 10 target functions, across 10 values of proportion output connectivity. In (**d**), 500 instances were averaged for each proportion output connectivity: 50 networks, 10 target functions. In (**e**), the average of 50 networks is shown for an example target function and $p_{\text {out}} = 0.5$. Error bands in (**d**, **e**) show the $95\%$ confidence interval.

Given that neuromodulation increased the stability of spike time coding, we were interested in whether population level activity could be used to “read out” stable temporal sequences. This may be understood, for example, as the network activity controlling a motor movement, or more generally, representing information in spike timing at the population level. To test this, we trained a linear readout on the spike data to produce an arbitrary 1-dimensional trajectory (a random walk, see Fig. 7e), referred to as the target function. In particular, we learned a set of output weights using ridge regression on the 1 ms binned spike data from the three learning pulses and recall simultaneously (see “Methods” section; cf. Fig. 1d). Using these learned weights, we then computed the predicted output on the recall trials and measured how well it matched the desired output, i.e. the target function, to test whether there is sufficient shared temporal information between learning and recall to store temporal sequences. We did this while sampling different proportions of the 1600 excitatory neurons, from 10 to 100% (see Fig. 7), and repeated this subsampling protocol for *only* core neurons (Supplementary Fig. S5) and *only* non-core neurons (Supplementary Fig. S6).

To assess whether consolidation had an effect on temporal sequence storage we first compared 10 s and 8 h recall. For this, we averaged over 10 different target functions and 10 subsampling rates (proportion output connectivity) and examined how temporal sequence storage (goodness of fit, $R^2$) depended on neuromodulator concentration and stimulation frequency at 10 s and 8 h recall (see Fig. 7). For recall after 10 s, as expected, there was no effect of neuromodulator. Target functions could be learned well, with a decrease in performance only for very low stimulation frequencies (Fig. 7a). For 8 h recall, that is after consolidation, there was a clear effect of neuromodulator concentration. For low levels of neuromodulation, the network activity was not able to support the storage of temporal sequences (yellow star, Fig. 7b), with a significant decrease in performance compared with 10 s recall (Mann-Whitney $U = 0$, $n_1=n_2=50$, $p < 0.0001$, two-tailed). With increasing levels of neuromodulation, temporal sequence storage improved, and for moderate to high neuromodulator levels, it was comparable to 10 s recall (see Fig. 7b, c). This suggests temporal sequences can only survive the transfer to the late phase when neuromodulator levels are high enough. For high neuromodulator concentrations, the shared temporal information between learning and 8 h recall was even very slightly above that for 10 s recall (blue star, Fig. 7a, b, Mann-Whitney $U = 1756$, $n_1=n_2=50$, $p = 0.0005$, two-tailed).

Further, by using our subsampling protocol we were able to evaluate how many output projections were required to learn an arbitrary output sequence (see Fig. 7d). This analysis sheds light on the number of neurons that participate in conveying temporal information and the extent to which the information they provide is independent from other neurons. For low neuromodulator levels, performance increased approximately linearly with the percent connectivity, maxing out at $R^2 \approx 0.85$. On the other hand, when neuromodulator levels were high, performance reached $R^2 \approx 0.85$ with only $50\%$ connectivity, i.e. requiring only half as many output connections, and $R^2 \approx 0.97$ for $100 \%$ connectivity (Fig. 7d). Thus, the number of output projections required to read out a temporal sequence from the network depends crucially on neuromodulator levels during consolidation.

Finally, we found that the improved storage of temporal sequences at higher levels of neuromodulation depends on neurons outside of the core assembly. Core assembly neurons alone were not sufficient to store temporal sequences (Supplementary Fig. S5). However, when only non-core neurons were sampled (Supplementary Fig. S6), the pattern of results was similar to the storage of temporal sequences when all neurons were sampled (Fig. 7). Thus, neuromodulator-dependent outgrowth of the assembly recruits neurons that support the storage of temporal sequences.

Taken together, these results show that high neuromodulator levels during consolidation give rise to shared temporal information in the firing patterns of the recurrent network between learning and recall, even when only half of the neurons are stimulated at recall 8 h later. The emergent temporal structure could, for example, be part of a motor sequence, an episodic memory, or it could simply code for some information like a smell, akin to “odor sequences” in the hippocampus^62,63^.

## Discussion

We have shown that neuromodulation can retroactively modify the neural code for a recent learning experience. For low levels of neuromodulation after learning, an input-defined rate code is transferred to the late phase and exhibits good recall performance 8 h later. For high levels of neuromodulation, instead, a self-organized rate code exhibits better recall performance. Looking deeper, we found that this self-organized rate code contains temporal structure encoded by spike timing, which gives rise to a temporal memory component. Furthermore, we found that in our model, the neuromodulator-dependent effect on coding is accompanied by a parallel effect on the consolidation of synaptic weights. In particular, while under low levels of neuromodulation STC only transfers core-internal synapses to the late phase, under higher levels of neuromodulation both core-internal and outgoing synapses are transferred. Importantly, we have shown that for a single learning event, the dominant type of neural coding given by the activity pattern at recall depends on the neuromodulator concentration during consolidation.

But why might a single network in the brain need to switch between rate coding and temporal coding *retroactively*? The critical point is that the utility of an event and the nature of its consequences are often not clear until after it is over, when reward or other task-related information becomes available (cf.^25,26,64^). Thus, both types of coding should be eligible during the early stages of learning a task, while task-specific neuromodulator release subsequently promotes a particular type of code to be preferentially consolidated into long-term memory. This is also consistent with the finding that dopamine in hippocampal CA1 after stimulation can retroactively modify the effect of a learning stimulus, even converting synaptic depression into potentiation^27,39^.

To explain the retroactive modification of coding, our model assumes that there should be mechanisms to flexibly release or maintain the appropriate amount of neuromodulator after an experience. This could happen via the type of activity pattern (e.g., tonic or phasic) in the neuromodulator-releasing brain area^65,66^ or via the mechanism of neuromodulator release into the target brain region (e.g., dopamine release via reverse transport through the norepinephrine transporter^67^). Furthermore, flexible release could be guided by the recruitment of different neuromodulatory systems. In particular, both the locus coeruleus (LC) and the ventral tegmental area (VTA) are known to release dopamine into the hippocampus, with LC also releasing norepinephrine. The dorsal hippocampus receives vastly more dopaminergic projections from the LC than from the VTA^68^, suggesting that activation of the LC should elicit higher neuromodulator concentrations in hippocampus. Considering this, our results would suggest that LC activation may enhance consolidation of memories with a temporal component while VTA should enhance the consolidation of non-temporal information. Interestingly, this agrees with a recent proposal^69^ suggesting that LC promotes consolidation of episodic memory, known to contain a temporal component, while VTA promotes consolidation of semantic memory, considered to lack a temporal component. While the authors suggest that VTA promotes semantic memory via synaptic and systems consolidation and LC promotes episodic memory via synaptic consolidation only, our results would instead suggest that the type of memory stored can be explained simply by the concentration of neuromodulator present during synaptic consolidation. Investigating the role of systems consolidation was, however, beyond the scope of our study. Duszkiewicz and colleagues^69^ additionally proposed that the VTA and LC neuromodulatory systems are recruited for different types of novelty processing (VTA when an event has similarities to past events, LC when the experience is distinct). Along these lines, our results may provide further evidence for the connection between synaptic tagging and behavioral tagging, where novelty-induced neuromodulation enables the transfer of rather unimportant episodic details to long-term memory (cf.^38,40^). In particular, our results on varied timing of the neuromodulator signal exhibit parallels to pertinent experiments. The extent to which our model can account for behavioral tagging may be investigated more deeply, for example, as it was done in a feed-forward network approach^43^.

Here, we investigated how neuromodulation as well as learning stimulation influence the functionality of memory representations. The principal difference in the impact of neuromodulation and learning stimulation is given by the nonlinear nature of the synaptic tag. Once the tag of a particular synapse has vanished, this synapse no longer undergoes stabilization through STC, even if neuromodulation is drastically increased. Learning stimulation, on the contrary, is able to modify the synaptic weight and thereby controls the lifetime of the synaptic tag, which defines the time window for STC (cf. Fig. 1b, c). The following thought experiment shall demonstrate why this difference matters. Consider learning stimulation that evokes the formation of a cell assembly with moderate outgrowth (as in the ‘moderate’ case in Fig. 2). Right after learning, the core of the assembly will exhibit strong internal weights, while the outgoing weights will mostly be of moderate strength. This corresponds to long lifetimes of the tags of core-internal synapses, and medium lifetimes of the tags of outgoing synapses. Hence, shortly after most of the tags of outgoing synapses have vanished, most core synapses will still be tagged. If neuromodulation is drastically increased at this point in time, the core of the assembly will undergo enhanced stabilization, while the outgrowth will not experience any more stabilization. Stimulation, for instance a second learning stimulus occurring after the initial one, or reactivation, for example during hippocampal replay, can affect any synapse at any time and therefore does not yield this type of selective retrograde modification. This prediction is supported by our simulation results on recall performance and the protein amount as a function of time (Fig. 4 and Supplementary Fig. S2).

The spatial and temporal profiles of neuromodulator release exist across multiple orders of magnitude^65^. Release can take place very locally with the substance being cleared up in milliseconds, however, it can also can also be widespread and last for hours. Mather and colleagues^50^ have suggested that spatially localized norepinephrine release in cortex and hippocampus triggers local protein synthesis to enhance memory consolidation in selected cell populations. In hippocampal CA2, selective release of neuromodulators has been suggested to prioritize important experiences for replay via neuromodulator-dependent plasticity (dependent e.g. on vasopressin, oxytocin, or substance P;^70,71^). Our results here suggest that widespread release coming after an experience can still selectively determine the neuronal subpopulation recruited to store a memory. Even more, the coding dynamics of this subpopulation are controlled by the amount of neuromodulator.

We have constrained our investigations here to the simplest possible situation that can shed light on the question how neuromodulator-dependent consolidation retroactively affects acquired information. Of course, in the brain, a host of neuromodulatory influences can be present simultaneously^64,72,73^. In addition to synaptic consolidation, they may also affect excitability^50,69^ and early-phase synaptic plasticity at the time of encoding^74–78^, which can serve to select a subpopulation of neurons for memory formation^50^ before undergoing neuromodulator-dependent consolidation. Even without strong electrical stimulation, neuromodulators can induce a slow-onset potentiation that lasts for hours^49,70,79,80^. Furthermore, for different brain regions, there can be different neuromodulatory requirements for STC, for example in hippocampal CA1 vs. CA2^80–83^. Interestingly, synaptic as well as behavioral tagging also depend on sex and change with age and neurodegeneration^84–86^. Finally, different molecules including receptor subunits, CaMKII, PKM$\zeta$, and actin have been suggested to affect synaptic plasticity either as plasticity-related proteins or as constituents of the synaptic tag, but the exact related mechanisms remain elusive^87–91^.

Our results, however, show by proof of principle that a generic mechanism with abstract neuromodulator, proteins, and synaptic tag can control the consolidation of synaptic subpopulations and thereby determine functional dynamics for the recall of long-term memory representations in recurrent spiking neural networks. We could relate these functional dynamics to rate codes and temporal sequences, which have been extensively treated in the literature^8,11,55,92^. Note that the abstract neuromodulator that we considered in this study could also represent effective modulation resulting from the combined effects of more than one neuromodulatory substance. Furthermore, while this study targeted networks holding a single cell assembly, our previous work^93^ has treated multiple cell assemblies with a similar model, and our provided simulation code^94^ can be readily used for multi-assembly investigations.

Previous experimental studies have shown that the presence of neuromodulators is necessary for synaptic memory consolidation. Here, we have provided a theoretical model that supports these findings and further makes predictions about the impact of *neuromodulator concentration*. Our modeling results lead to two main predictions: (i) by influencing the synthesis of plasticity-related proteins, neuromodulator concentration may serve to retroactively select a subset of synaptic connections in the network to become stabilized; (ii) the neuromodulation-dependent synaptic changes influence spiking dynamics, ultimately controlling which type of neural code is used to store information about an experienced stimulus. These predictions have, to the best of our knowledge, not yet been tested experimentally. Future experiments could aim to stimulate a subset of neurons while recording from a larger area of cortex or hippocampus. Variation of neuromodulator concentrations across trials, both with and without the presence of protein synthesis inhibitors, can then serve to test our predictions. That is, higher neuromodulator concentrations should promote strengthening of synapses from the stimulated neurons to non-stimulated neurons in a protein synthesis-dependent manner, and this should increase spike time stability (see “Methods” section). To determine whether concentration-dependent effects exist in naturalistic settings, it would be equally important to assess whether different amounts of neuromodulator are released to store different types of memory (in particular, episodic vs. semantic memory; cf.^69^). In order to optimally store memories, these features may be crucial for a brain to possess.

## Methods

The model, the stimulation protocols, and the measures used in this study are described in this section. Unless stated elsewhere, the parameters that we used are shown in Tables 1 and 2.

### Network model

To simulate the dynamics of long-term memory representations, we use the network model of memory consolidation with synaptic tagging and capture from our previous work^44^ (for more details, please see that paper). The network has hippocampal/neocortical characteristics regarding firing rate and connectivity and comprises spiking neurons and synapses with detailed plasticity features. As we use leaky integrate-and-fire neurons, the membrane potential of neuron *i* is given by the following equation (cf.^95^):

1 $$\begin{aligned} \tau _{\text {m}}\frac{dV_i(t)}{dt} = V^{\text {rev}} - V_i(t) + \, V_i^{\text {PSP}}(t) + V_{i}^{\text {bg}}(t) + V_{i}^{\text {stim}}(t) \end{aligned}$$

where $V^{\text {rev}}$ is the reversal potential, $\tau _{\text {m}}$ is the membrane time constant, $R_{\text {m}}$ is the membrane resistance, and $V^{\text {bg}}(t)$ and $V^{\text {stim}}(t)$ are the external background and external stimulus contributions, respectively. The synaptic weights and presynaptic spike times are given by $w_{ji}$ and $t^k_j$, respectively, yielding the postsynaptic potentials evoked by neurons from within the network:

2 $$\begin{aligned} V_{i}^{\text {PSP}}(t) := \sum \limits _j\sum \limits _{t^k_j} w_{ji} \cdot \exp \left( -(t-t^k_j -t_{\text {ax,delay}})/\tau _{\text {syn}}\right) \Theta (t-t^k_j -t_{\text {ax,delay}}), \end{aligned}$$

where $t_{\text {ax,delay}}$ is the axonal delay time, $\tau _{\text {syn}}$ is the synaptic time constant, and $\Theta (\cdot )$ is the Heaviside step function.

If the membrane potential $V_i$ crosses the threshold *V*th, a spike is generated and its time of occurrence is stored to serve as input to other neurons and to be used for computation of firing rates and temporal traces. Subsequently, the membrane potential is reset to $V^{\text {reset}}$, and remains fixed at this value for the refractory period $t_{\text {ref}}$. In the absence of learning and recall stimulation, only background noise causes additional fluctuation of the membrane potential as described by the following Ornstein-Uhlenbeck process:

3 $$\begin{aligned} \tau _{\text {syn}}\frac{dV_{i}^{\text {bg}}(t)}{dt} = - V_{i}^{\text {bg}}(t) + R_{\text {m}}\left( I^0 + \sigma _{\text {wn}}\cdot \Gamma _i(t)\right) \end{aligned}$$

with mean current $I^0$, white-noise standard deviation $\sigma _{\text {wn}}$, and Gaussian white noise $\Gamma _i(t)$ with mean zero^96^. Since the power spectrum of the Ornstein-Uhlenbeck process resembles that of fluctuating input from a large presynaptic neuronal population in the cortex^97^, it accounts for synaptic inputs from outside our network. Besides the background noise, we also model the postsynaptic potentials evoked by learning and recall stimulation by an Ornstein-Uhlenbeck process:

4 $$\begin{aligned} \tau _{\text {syn}}\frac{dV_{i}^{\text {stim}}(t)}{dt} = - V_{i}^{\text {stim}}(t) + \left( N_{\text {stim}}\cdot f_{\text {stim}} +\sqrt{ N_{\text {stim}}\cdot f_{\text {stim}}}\cdot \Gamma _i(t)\right) \cdot 1\,{\text {s}} \cdot h_0 . \end{aligned}$$

Here, mean and standard deviation of the process are defined by putative input spikes to the stimulated neuron, conveyed by $N_{\text {stim}}$ neurons at the frequency $f_{\text {stim}}$ through synapses of weight $h_0$^8,44,96^.

Our neural network consists of 1600 excitatory and 400 inhibitory neurons, the ratio between excitatory and inhibitory neurons being typical for cortical and hippocampal networks^98^. A spike occurring in neuron *j* is transmitted to neuron *i* (cf. Eq. 1) if there is a connection from *j* to *i*. The probability of connection across our whole network is 10%, which is a reasonable approximation for hippocampal and neocortical networks^99^. The postsynaptic current that is evoked in neuron *i* by a presynaptic spike from neuron *j* depends on the weight of the corresponding synapse, which is given by:

5 $$\begin{aligned} w_{ji} = {\left\{ \begin{array}{ll} \quad h_{ji} + h_0 \cdot z_{ji} &{} {\text {for E}}\rightarrow E, \\ \quad w_{\text {ei}} &{} {\text {for E}}\rightarrow I, \\ \quad w_{\text {ie}} &{} {\text {for I}}\rightarrow E, \\ \quad w_{\text {ii}} &{} {\text {for I}} \rightarrow I, \end{array}\right. } \end{aligned}$$

where E relates to excitatory and I relates to inhibitory neurons. While the synaptic connections involving inhibitory neurons are constant, the total synaptic weight $w_{ji}$ of E$\rightarrow$E connections consists of two variable contributions, which is a critical feature of STC mechanisms. The first contribution is the early-phase weight $h_{ji}$ and the second is the late-phase weight $z_{ji}$. We used $h_0$ to normalize *z*, such that it has the same dimension as *h*. This factor is in accordance with experimental data (cf. ^44,54^).

The early-phase weight is described by the following differential equation:

6 $$\begin{aligned} \tau _h \frac{dh_{ji}(t)}{dt} = 0.1\,(h_0 - h_{ji}(t)) + \gamma _{\text {p}}(10\,{\text {mV}}-h_{ji}(t)) \cdot \Theta [c_{ji}(t)-\theta _{\text {p}}] \nonumber \\ - \gamma _{\text {d}}h_{ji}(t)\cdot \Theta [c_{ji}(t)-\theta _{\text {d}}] + \xi (t), \end{aligned}$$

where $\tau _h$ is a time constant, $c_{ji}(t)$ is the calcium concentration at the postsynaptic site. Early-phase LTP occurs with rate $\gamma _{\text {p}}$ if the calcium concentration is above the threshold $\theta _{\text {p}}$, and early-phase LTD occurs with rate $\gamma _{\text {d}}$ if the calcium concentration is above the threshold $\theta _{\text {d}}$. In addition, a noise term is added if LTD or LTP occurs: $\xi (t) = \sqrt{\tau _h \left[ \Theta \left( c_{ji}(t)-\theta _{\text {p}}\right) + \Theta \left( c_{ji}(t)-\theta _{\text {d}}\right) \right] }\,\sigma _{\text {pl}}\,\Gamma (t)$, with a scaling factor $\sigma _{\text {pl}}$ and Gaussian white noise $\Gamma (t)$ with mean zero and variance 1/*dt*.

The calcium-based model of early-phase plasticity is based on the calcium-driven plasticity model by^61^, using parameters fitted on hippocampal slice data^100^, and corrected by a factor of 0.6 to account for in vivo conditions^101^. The relaxation term was introduced by^54^ to account for synaptic tagging and capture, along with the late-phase weight dynamics described below.

The calcium concentration depends on pre- and postsynaptic spikes at times $t^n_j$ and $t^m_i$, and is described by the following equation:

7 $$\begin{aligned} \frac{dc_{ji}(t)}{dt} = -\frac{c_{ji}(t)}{\tau _{\text {c}}} + c_{\text {pre}}\,\sum \limits _{n}\delta (t-t^n_j-t_{\text {c,delay}}) + c_{\text {post}}\,\sum \limits _{m}\delta (t-t^m_i), \end{aligned}$$

where $\tau _c$ is a time constant, $c_{\text {pre}}$ is the increase in calcium evoked by presynaptic spikes, $c_{\text {post}}$ is the increase in calcium by postsynaptic spikes, $t_{\text {c,delay}}$ is a delay for presynaptic signals, and $\delta (\cdot )$ is the Dirac delta distribution.

The late-phase synaptic weight depends on the early-phase weight and is given by^54^:

8 $$\begin{aligned} {\tau _{z}}\frac{dz_{ji}(t)}{dt} =&\quad p_i(t)\cdot (1-z_{ji}(t))\cdot \Theta [(h_{ji}(t)-h_0)-\theta _{\text {tag}}]\nonumber \\&- p_i(t)\cdot (z_{ji} + 0.5)\cdot \Theta [(h_0-h_{ji}(t))-\theta _{\text {tag}}], \end{aligned}$$

where $\tau _z$ is a time constant, $p_i(t)$ is the amount of plasticity-related proteins, and $\theta _{\text {tag}}$ is the tagging threshold. Both late-phase LTP and late-phase LTD depend on the amount of plasticity-related proteins and on the presence of the synaptic tag. The synapse is tagged if the change in early-phase weight $\left| h_{ji}(t)-h_0\right|$ exceeds the tagging threshold $\theta _{\text {tag}}$. The late-phase weight changes when the synapse is tagged and plasticity-related proteins are abundant ($p_i(t) > 0$).

New plasticity-related proteins are synthesized if the sum of changes in early-phase weight across the whole neuron exceeds the protein synthesis threshold $\theta _{\text {pro}}$^52^:

9 $$\begin{aligned} \tau _p \frac{dp_i(t)}{dt} = -p_i(t) + \alpha \, \Theta \left[ \left( \sum \limits _j \left| h_{ji}(t)-h_0\right| \right) -\theta _{\text {pro}}\right] . \end{aligned}$$

While we previously^44^ used a fixed protein synthesis threshold, we consider it variable, in order to model the impact of neuromodulation. The protein synthesis threshold depends on the concentration of neuromodulator NM in the following way^52^:

10 $$\begin{aligned} \theta _{\text {pro}}( NM ) = \frac{1}{ NM +0.001}. \end{aligned}$$

### Protocol to learn and recall spatial patterns

Initially, we let the activity settle for 10.0 s, before applying the learning protocol. The learning protocol included three stimulus pulses, each lasting for 0.1 s, applied to 150 neurons in the network (see Fig. 1d). The stimulus pulses were separated by breaks of 0.4 s. During the pulses, the stimulus current, described by Eq. (4), enters the neuronal membrane potential, given by Eq. (1).

We applied a recall stimulus either 10 s or 8 h after the end of the learning stimulus. The recall stimulus consisted of one pulse, lasting for 0.1 s, to half of the neurons that had earlier received the learning stimulus. The stimulus current for the recall stimulus was also modeled by Eq. (4).

The network simulations that we needed to perform for this study were computationally extremely demanding. Although it was very helpful that we could use the computing cluster of the Gesellschaft für wissenschaftliche Datenverarbeitung mbH Göttingen (GWDG) and although we used compiled C++ code, this study would still not have been feasible if we had had to run our spiking network simulations in full detail. Thus, in those simulations with recall stimulation after 8 h, we refrained from computing the spiking dynamics during the consolidation phase in between learning and recall stimulus and just computed the late-phase dynamics and the exponential decay of the early-phase weights. We have shown in^44^ and in^102^ that neglecting such sparsely occurring spikes does not change the weight dynamics of a synapse.

### Measuring spatial recall

We used two measures to determine the performance in recalling a spatial pattern (note that these measures were also used in^44^).

The input-defined measure *Q* describes the degree to which the pattern defined by the learning stimulus (which can be described in short by “150 neurons ‘on’, the rest ‘off’”) is completed upon activation of half of the originally stimulated neurons. To compute this, the excitatory population is divided into three subpopulations: assembly neurons that are stimulated by both recall and learning stimulus (“as”), assembly neurons that are not stimulated by recall but were stimulated by learning stimulus (“ans”), and control neurons that are not stimulated by neither recall nor learning stimulus (“ctrl”)—see Fig. 3g. The function $\nu \left( t, n\right)$ describes the firing rate of a given neuron *n* at a given time *t*, computed using a sliding window of $0.5\,{\text {s}}$. Thereby, the mean firing rates in the three subpopulations upon 10 s and 8 h recall are given by $\bar{\nu }_{\text {as}}$, $\bar{\nu }_{\text {ans}}$, and $\bar{\nu }_{\text {ctrl}}$ at $t_{\text {recall}}=20.1\,{\text {s}}$ and $t_{\text {recall}}=28810.1\,{\text {s}}$, respectively. Based on these mean activities, the quality of input-defined recall is computed by the following equation:

11 $$\begin{aligned} Q := \frac{\bar{\nu }_{\text {ans}} - \bar{\nu }_{\text {ctrl}}}{\bar{\nu }_{\text {as}}}. \end{aligned}$$

Perfect pattern completion of the input-defined pattern would thus be achieved for $Q=1$ (which is hard to achieve in a non-attractor system), while $Q=0$ describes the case with no pattern completion at all.

In addition to measuring the recall of the input-defined pattern, we measured the performance of recalling a self-organized pattern. To do this, we considered the mutual information $MI_{\nu }$ between the distribution of firing rates at the end of recall and at the end of learning. Specifically, this is calculated from the entropy at time $t_{\text {learn}}=11.0$ s (during learning), the entropy at time $t_{\text {recall}}=20.1$ s or $t_{\text {recall}}=28810.1$ s (during 10 s or 8 h recall), and the joint entropy between both:

12 $$\begin{aligned} { MI }_{\nu } := H\left( \nu \left( t=t_{\text {learn}}, n\right) \right) + H\left( \nu \left( t=t_{\text {recall}}, n\right) \right) - H\left( \nu \left( t=t_{\text {learn}}, n\right) , \nu \left( t=t_{\text {recall}}, n\right) \right) . \end{aligned}$$

### Dimensionality estimation

We aimed to estimate the amount of temporal information available to a downstream readout from the spiking activity of the 1600 excitatory neurons during recall both at 10 s and 8 h. For this, we estimated the dimensionality of the spike data using principal component analysis (PCA). The data was first binned into 1 ms bins before we applied PCA to the binned spike data from the 10 s and 8 h recall trials using sklearn.decomposition.PCA from Python’s scikit-learn library. The data is stored in a matrix containing ones when a neuron spiked, and otherwise zeros (despite binning given the refractory period a neuron can only fire one spike in a 1 ms bin). Its dimension is 1600 excitatory neurons by 100 time points (100 ms stimulus with 1 ms temporal bins). To estimate dimensionality we computed the number of principal components required to retain 70% of the variance. This was done for each neuromodulator amount and learning stimulus frequency for 50 randomly initialized networks. Averaging over the data from the 50 networks leads to the matrices for 10 s and 8 h recall shown in Fig. 5. Mann-Whitney *U* tests were used to compare dimensionality of neural activity at 8 h recall with 10 s recall for none, low, moderate, and high neuromodulator levels using scipy.stats.mannwhitneyu from Python’s SciPy library. For Supplementary Fig. S4, we repeated this procedure with 2 ms and 4 ms bins.

### Stability of spike times

To measure the stability of spike times we compared the spike patterns during the last (the third) pulse of the learning stimulus and 8 h recall. We binned the spike trains into 1 ms bins, for a total of 100 bins. For each neuron, we then counted the number of bins in which a spike occurred during both learning and recall. This count was divided by the total number of spikes during the learning stimulus to give a percentage of correctly matching spike times at recall. For each of the 50 network simulations, we averaged the stability score across neurons.

To generate shuffled data, for each neuron we randomly shuffled the time bins for both learning and recall. For example, through shuffling the time bin order [0, 1, 2, 3, ..., 100] might become [16, 3, 89, 21, ..., 7]. This was done independently for each neuron. Again, stability was measured for the shuffled data in the same way as for the real data. The resulting distributions were tested for normality (none: real, $p = 0.005$; shuffled, $p = 0.058$; low: real, $p = 0.47$; shuffled, $p = 0.97$; moderate: real, $p = 0.62$; shuffled, $p = 0.91$; high: real, $p = 0.16$; shuffled, $p = 0.44$, Shapiro-Wilk test) and then a two-way ANOVA was applied to test the effect of neuromodulator concentration and real/shuffled on spike time stability. Post-hoc two-tailed independent samples t-tests were applied to investigate the main effects.

For Fig. 6c, instead of averaging over neurons within a network simulation, we considered the stability score for each individual neuron outside of the core assembly (1450 neurons $\times \,50$ network simulations = 72,500 neurons). For each of these neurons, we computed the mean late phase weight of synaptic connections to that neuron from the core assembly. We did this for low, moderate, and high neuromodulator levels and computed Spearman’s rank correlation for spike time stability versus mean late phase weight. We generated scatter plots and computed least squares linear fits to visualize the relationship between spike time stability and synaptic weights (Fig. 6c).

### Temporal sequence learning

To test whether temporal structure present during learning is also present during recall, we trained the network to produce temporal sequences. We binned the data into 1 ms bins and generated 10 Gaussian random walks given by

$$\begin{aligned} y[i+1] = y[i] + \mathcal {N}(0, 1) \end{aligned}$$

with $y[0] = 1$ and length of 100 time bins. We then computed linear least squares with L2 regularization (using sklearn.linear_model.Ridge), which minimizes the objective function

$$\begin{aligned} || y - X w ||^2_2 + \alpha || w ||^2_2 \end{aligned}$$

where *y* is the target function and *X* is the data matrix of dimension (number of samples, number of features). Here the target is our random walk and the data are the binned spike rasters (transposed). In practice, to train the model, *y* and *X* are concatenated over the three learning trials and one recall trial, such that $y \in {\mathbb {R}}^{400}$, with 400 time bins, and $X \in {\mathbb {R}}^{400 \, \times \, (p_{\text {out}}\cdot 1600 + 1)}$ again with 400 time bins and $p_{\text {out}} \cdot 1600$ neurons plus one dimension for the intercept. The output weight vector is $w \in {\mathbb {R}}^{p_{\text {out}}\cdot 1600 + 1}$, a weight for each neuron plus one dimension for the intercept. Here $\alpha = 0.1$ is the regularization parameter which acts to keep the weights *w* from becoming too large. For each of the 10 random walk target functions we used 10 different values of $p_{\text {out}}$, namely $p_{\text {out}} \in \{0.1, 0.2, 0.3, 0.4, 0.5, 0.6, 0.7, 0.8, 0.9, 1.0\}$. The linear regression was computed in this way for each of the 10 s and 8 h recalls. Then, the resulting models were used to predict the target function only based on the data from the appropriate recall trial. This was repeated for each neuromodulator amount and learning stimulation frequency for each of the 50 networks.

To produce Fig. 7a–c, we averaged over the $R^2$ scores for the predictions on the 10 target functions, 10% output connectivities, and 50 networks. For Fig. 7d we computed $R^2$ as a function of proportion output connectivity averaging over the 10 target functions and 50 networks and in Fig. 7e we show results for an example target function. For Supplementary Fig. S5 we repeated the above for neurons from the core assembly only and for Supplementary Fig. S6 for non-core neurons only.

### Software

We used C++ in the ISO 2011 standard to implement our simulations. To compile and link the code, we employed g++ with boost. For data analysis and the creation of plots we used Python, in particular NumPy, scikit-learn, SciPy, Matplotlib, seaborn, as well as gnuplot. Our code has been released under the Apache-2.0 license and can be retrieved freely^94^.

**Table 1:** Parameters for neuron and static network dynamics. Values were used as given in this table, unless stated otherwise.

Symbol	Value	Description	Refs.
$\Delta t$	0.2 ms	Duration of one time step for numerical computation	This study
$\tau _{\text {m}}$	10 ms	Membrane time constant	^8,95^
$\tau _{\text {syn}}$	5 ms	Synaptic time constant, also for external input current	^8,95,103^
$t_{\text {ax,delay}}$	3 ms	Axonal spike delay	^95,104^
$t_{\text {ref}}$	2 ms	Duration of the refractory period	^8,105^
$R_{\text {m}}$	10 M$\Omega$	Membrane resistance	^8^
$V^{\text {rev}}$	−65 mV	Reversal (equilibrium) potential	^8^
$V^{\text {reset}}$	−70 mV	Reset potential	^8^
*V*th	−55 mV	Threshold potential to be crossed for spiking	^8^
$I^0$	0.15 nA	Mean of the external background current	^44^
$\sigma _{\text {wn}}$	0.05 nA s^1/2^	Standard deviation for Gaussian noise in the background current	^44^
$N_{\text {e}}$	1600	Number of neurons in the excitatory population	^44^
$N_{\text {i}}$	400	Number of neurons in the inhibitory population	^44^
$p_{\text {c}}$	0.1	Probability of a connection existing between two neurons	^99^
$h_0$	4.20075 mV	Initial excitatory$\rightarrow$excitatory coupling strength	^44,54^
$w_{\text {ei}}$	$2\,h_0$	Excitatory$\rightarrow$inhibitory coupling strength	^44^
$w_{\text {ie}}$	$4\,h_0$	Excitatory$\rightarrow$inhibitory coupling strength	^44^
$w_{\text {ii}}$	$4\,h_0$	Inhibitory$\rightarrow$inhibitory coupling strength	^44^
$f_{\text {recall}}$	100 Hz	Frequency of recall stimulation	^54,59^
$N_{\text {stim}}$	4	Number of input neurons for stimulation	This study
*r*	0.5	Fraction of assembly neurons that are stimulated to trigger recall	^44^

**Table 2:** Parameters for synaptic plasticity.

Symbol	Value	Description	Refs.
$t_{\text {c,delay}}$	0.0188 s	Delay of postsynaptic calcium influx after presynaptic spike	^61^
$c_{\text {pre}}$	0.6	Presynaptic calcium contribution, in vivo adjusted	^54,61,101^
$c_{\text {post}}$	0.1655	Postsynaptic calcium contribution, in vivo adjusted	^54,61,101^
$\tau _c$	0.0488 s	Calcium time constant	^54,61^
$\tau _h$	688.4 s	Early-phase time constant	^54,61^
$\tau _p$	60 min	Protein time constant	^52,54^
$\tau _{z}$	$60\,{\text {min}}$	Late-phase time constant	^52,54^
$\gamma _{\text {p}}$	1645.6	Potentiation rate	^54,61^
$\gamma _{\text {d}}$	313.1	Depression rate	^54,61^
$\theta _{\text {p}}$	3	Calcium threshold for potentiation	^54^
$\theta _{\text {d}}$	1.2	Calcium threshold for depression	^54^
$\sigma _{\text {pl}}$	2.90436 mV	Standard deviation for plasticity fluctuations	^54,61^
$\alpha$	1	Protein synthesis rate	^52,54^
$\theta _{\text {tag}}$	0.840149 mV	Tagging threshold	^54^

Values were used as given in this table, unless stated otherwise.

## Supplementary Information

Supplementary Information.

## Data Availability

We have released^94^ our simulation code, analysis scripts, and an interactive notebook, which enable reproduction of all results presented in this study. Furthermore, we provide partially processed data to readily use the interactive notebook, without previous running of simulations and analysis scripts, at https://doi.org/10.5281/zenodo.6981746. All datasets used and/or analyzed during the current study are available from the corresponding authors on reasonable request.
