# Supplementary material for: Neuromodulator-dependent synaptic tagging and capture retroactively controls neural coding in spiking neural networks
Source: Sci Rep. 2022 Oct 22;12:17772. doi: 10.1038/s41598-022-22430-7 (PMC9588040; doi:10.1038/s41598-022-22430-7)
Supplement: Supplementary file 1 — Supplementary Information. [file 41598_2022_22430_MOESM1_ESM.pdf]

Supplementary Information

Neuromodulator-dependent synaptic tagging and capture retroactively controls neural coding in spiking neural networks

Andrew B. Lehr [*,1,2,3], Jannik Luboeinski [*,1,2,3], Christian Tetzlaff [1,2,3]

[*]These authors contributed equally.
[1]Department of Computational Neuroscience, III. Institute of Physics – Biophysics, University of Göttingen, Göttingen, Germany
[2]Bernstein Center for Computational Neuroscience, Göttingen, Germany
[3]Department of Computational Synaptic Physiology, University Medical Center Göttingen, Germany

2022-08-11

Supplementary Figures

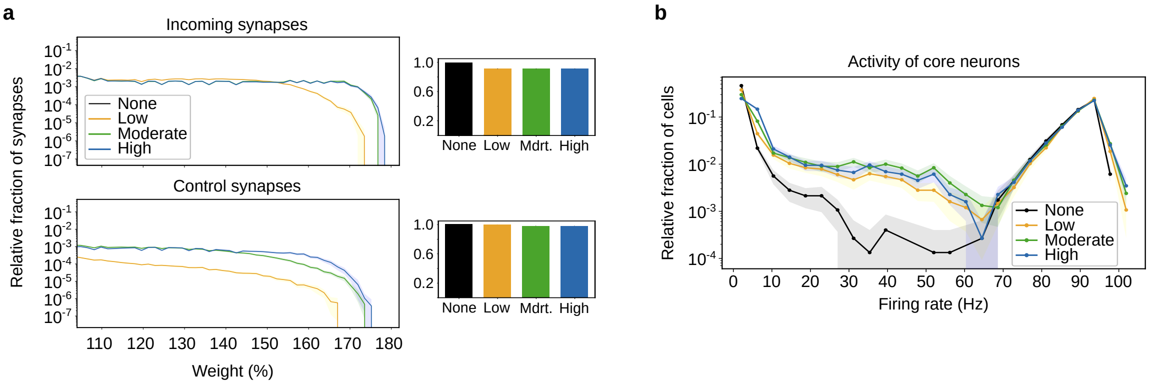

Supplementary Figure S1: **Further characteristics of the impact of neuromodulation on consolidation of a cell assembly (related to Figure 2 in main article).** **(a)** Distribution of the weights of incoming and control synapses at recall after 8 h for 60 Hz learning stimulation, averaged via bins of $\sim 1.64\%$. Bar plots on the right show the fraction of synapses that did not undergo substantial late-phase potentiation ($< 3.26\%$ potentiation, first two bins). **(b)** Distribution of the firing rates of the excitatory neurons inside the core assembly during recall 8 h after learning. Averaged via bins of 4.16 Hz across 50 networks.

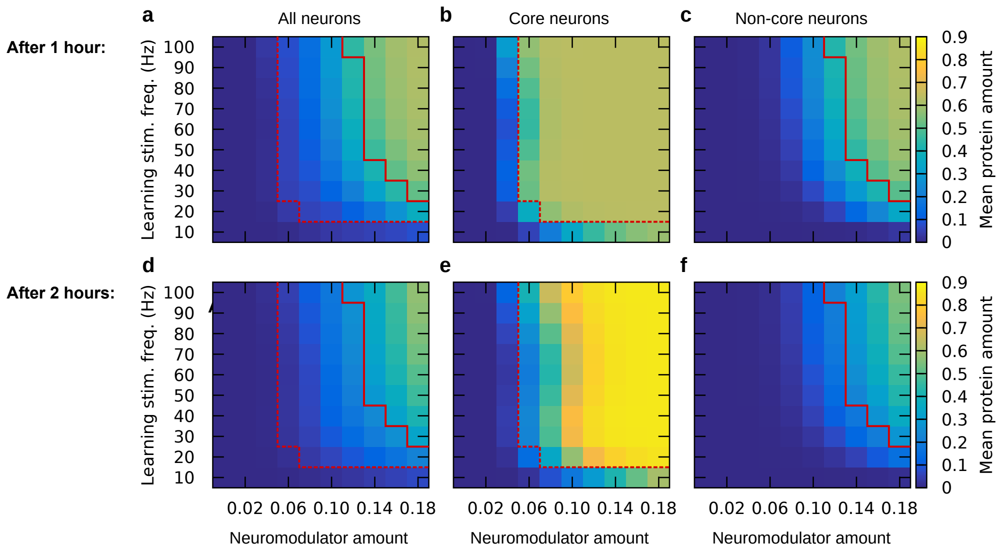

Supplementary Figure S2: **Mean protein amount in postsynaptic neurons.** **(a)** One hour after learning, mean of all excitatory neurons; **(b)** one hour after learning, mean of all core neurons; **(c)** one hour after learning, mean of all non-core neurons; **(d)** two hours after learning, mean of all excitatory neurons; **(e)** two hours after learning, mean of all core neurons; **(f)** two hours after learning, mean of all non-core neurons. All data were averaged over 50 networks.

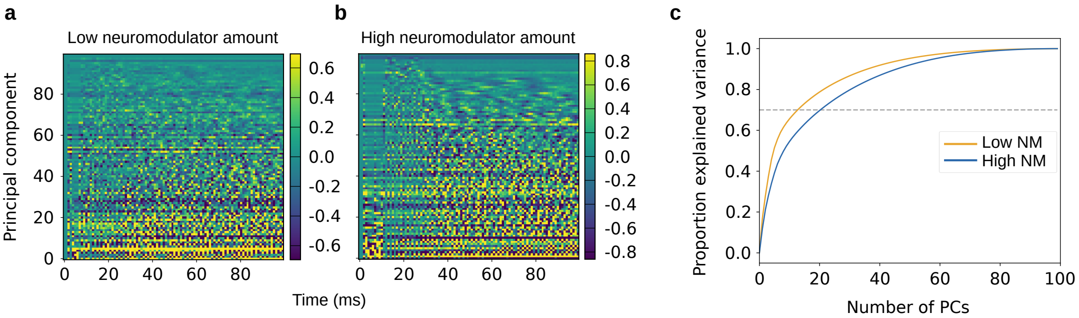

Supplementary Figure S3: **Principal components and dimensionality (related to Figure 5 in main article).** Examples of the transformed data (projection onto principal components) at 8h-recall for **(a)** low and **(b)** high neuromodulator levels, for visualization purposes clipped at the standard deviation ($\sigma_{\text{low}} = 0.69$ and $\sigma_{\text{high}} = 0.86$, $n = 10000$, 100 principal components $\times$ 100 time bins). **(c)** The cumulative variance plot for low (yellow) and high (blue) neuromodulator conditions (stars in Fig. 5a,b) at 8h-recall, averaged over 50 networks, error bands 95% confidence interval. Gray dashed line shows 70% of variance.

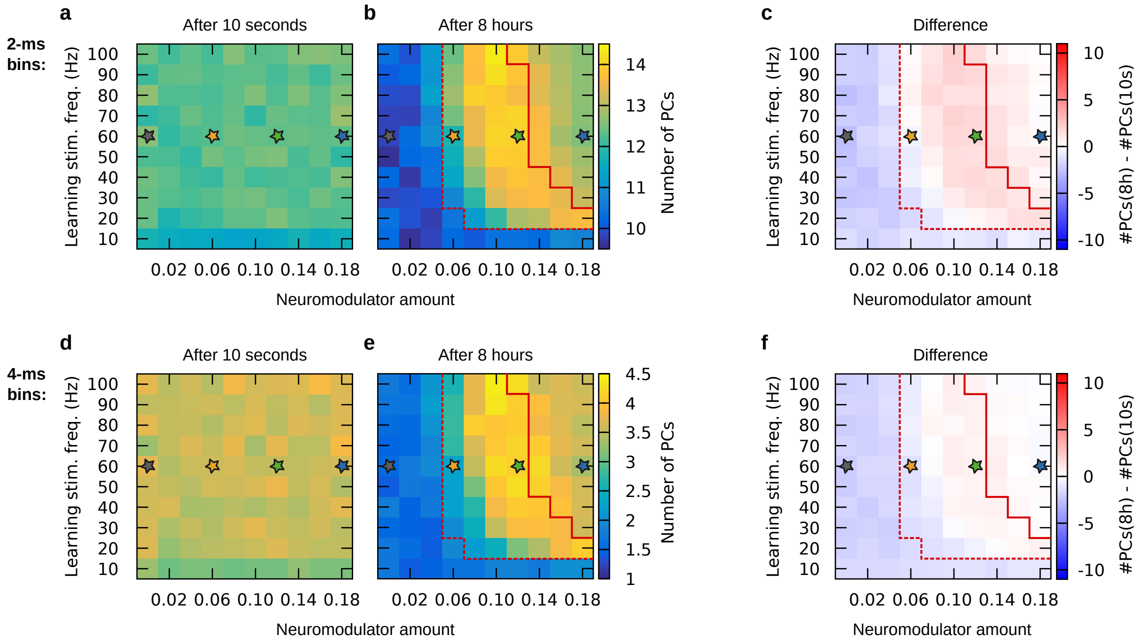

Supplementary Figure S4: **Dimensionality of neuronal activity upon recall, obtained with different time bins (related to Figure 5 in main article).** Number of principal components (PCs) required to explain 70% of variance in 2 ms-/4 ms-binned spike data during **(a,d)** 10s-recall and **(b,e)** 8h-recall. **(c,f)** The difference between 10s-recall and 8h-recall. Data were averaged over 50 networks (a,b,d,e) before being subtracted (c,f).

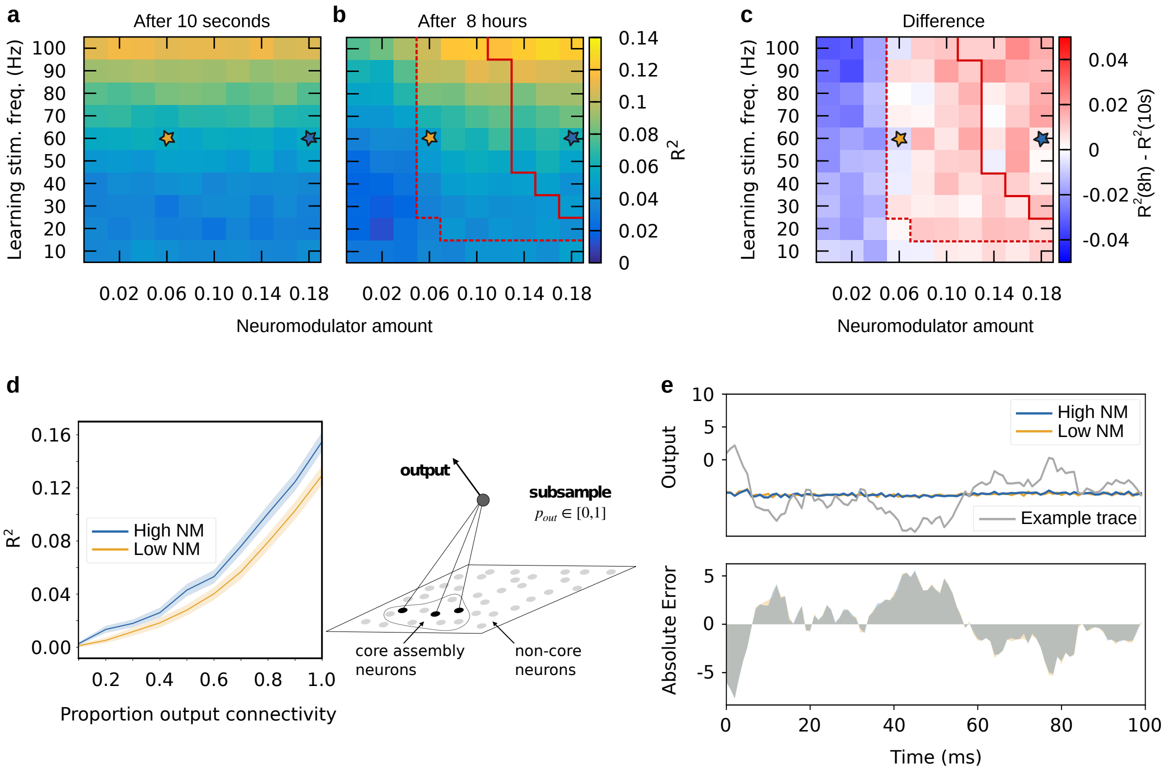

Supplementary Figure S5: **Core assembly is not enough to enable stable read out of temporal sequences (related to Figure 7 in main article).** The goodness of fit (R^2) of the regularized linear regression model is shown for recall **(a)** 10 s and **(b)** 8 h after learning. Yellow star represents low neuromodulator condition, blue star high neuromodulator. **(c)** The impact of neuromodulator-dependent STC on the shared temporal information during learning and recall is measured as the difference between the 8 h and 10 s results. **(d)** The goodness of fit is shown for high (blue) and low (yellow) across different percent output connectivities. A depiction of the subsampling process is shown on the right. **(e)** The predicted trajectory at 8 h recall in the low (yellow) and high (blue) neuromodulator conditions are shown for one example target function, gray. The bottom panel shows the absolute error (with sign). Data in (a,b,c) were averaged over 5000 instances: 50 networks with 10 target functions, across 10 values of output connectivity. In (d), 500 instances were averaged for each percent connectivity: 50 networks, 10 target functions. In (e), the average of 50 networks is shown for an example target function and $p_{\text{out}} = 0.5$. Error bands in (d,e) show the 95% confidence interval.

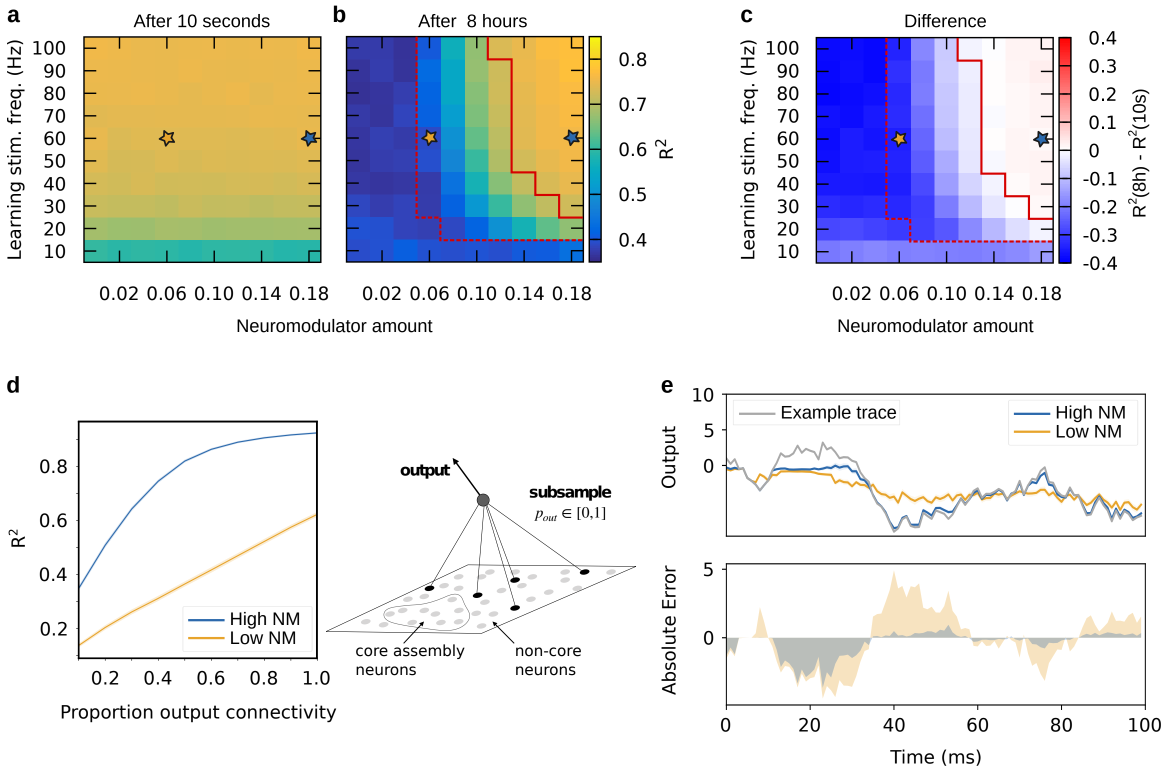

Supplementary Figure S6: **Non-core neurons enable good read out of temporal sequences (related to Figure 7 in main article).** The goodness of fit (R^2) of the regularized linear regression model is shown for recall **(a)** 10 s and **(b)** 8 h after learning. Yellow star represents low neuromodulator condition, blue star high neuromodulator. **(c)** The impact of neuromodulator-dependent STC on the shared temporal information during learning and recall is measured as the difference between the 8 h and 10 s results. **(d)** The goodness of fit is shown for high (blue) and low (yellow) across different percent output connectivities. A depiction of the subsampling process is shown on the right. **(e)** The predicted trajectory at 8 h recall in the low (yellow) and high (blue) neuromodulator conditions are shown for one example target function, gray. The bottom panel shows the absolute error (with sign). Data in (a,b,c) were averaged over 5000 instances: 50 networks with 10 target functions, across 10 values of output connectivity. In (d), 500 instances were averaged for each percent connectivity: 50 networks, 10 target functions. In (e), the average of 50 networks is shown for an example target function and $p_{\text{out}} = 0.5$. Error bands in (d,e) show the 95% confidence interval.

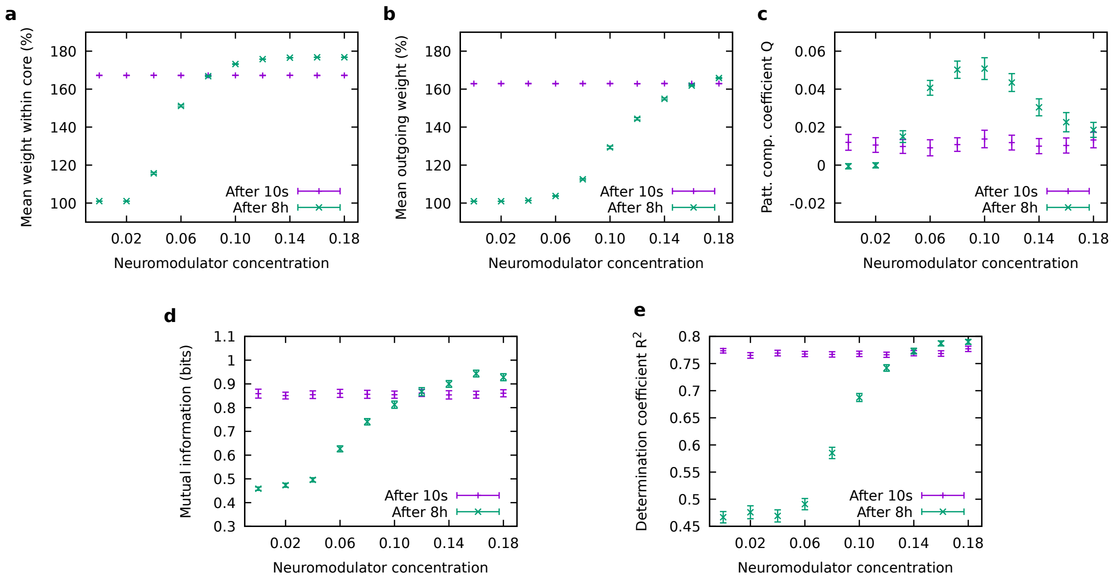

Supplementary Figure S7: **Synaptic weight and memory recall performance as a function of the neuromodulator amount (related to Figures 2, 3, 7 in main article).** (a) Mean weight of the synapses within the cell assembly core, 10 s and 8 h after learning. (b) Mean weight of the outgoing synapses from the cell assembly core to the rest of the excitatory population, 10 s and 8 h after learning. (c-e) Recall performance 10 s and 8 h after learning, (c) for an input-defined pattern, (d) for a self-organized pattern, (e) for the linear readout of a temporal trace (subsampled from all neurons in the network). For all panels, neuromodulation lasted for the whole duration of the simulation. Frequency of the learning stimulation was 60 Hz. Values were averaged over 50 networks; error bars show the 95% confidence interval.

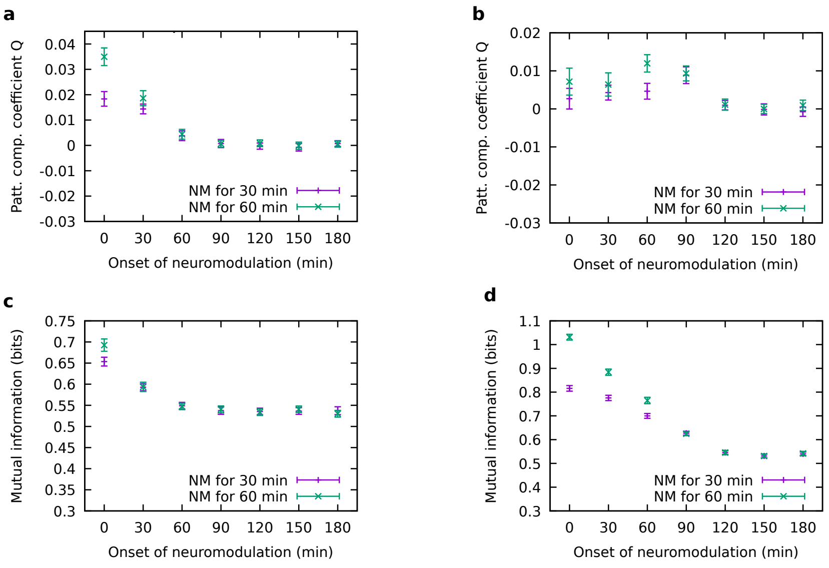

Supplementary Figure S8: **Memory recall performance as a function of the neuromodulator timing (related to Figure 4 in main article).** Recall performance after 8h with neuromodulation lasting for either 30 min or 60 min: (a) Weak neuromodulation (0.06), recall of an input-defined pattern; (b) strong neuromodulation (0.18), recall of an input-defined pattern; (c) weak neuromodulation (0.06), recall of a self-organized pattern; (d) strong neuromodulation (0.18), recall of a self-organized pattern. Frequency of the learning stimulation was 60 Hz. Values were averaged over 50 networks; error bars show the 95% confidence interval.

Supplementary Tables

bin size	neuromodulator	mean 10s	mean 8h	mean 8h - mean 10s	mann whitney u	p-value	sig. at α = 0.001
1ms	none vs. none	19.62	9.22	-10.4	U=0.0	p=3.42e-18	*
1ms	low vs. low	18.86	13.2	-5.66	U=4.5	p=6.22e-18	*
1ms	mod vs. mod	19.3	21.16	1.86	U=2040.5	p=3.18e-08	*
1ms	high vs. high	19.48	20.76	1.28	U=1783.5	p=1.82e-04	*

Supplementary Table S1: **Comparison of dimensionality at 10s- vs. 8h-recall for 1ms bins (related to Figure 5 in main article).** Table shows the results of Mann-Whitney U tests as well as relevant means, mean differences, and p-values. None, low, moderate, and high correspond to gray, yellow, green, and blue stars in Figure 5 in main article. Sample size is 50 network simulations per condition.

bin size	neuromodulator	mean 10s	mean 8h	mean 8h - mean 10s	mann whitney u	p-value	sig. at α = 0.001
2ms	none vs. none	12.6	9.82	-2.78	U=309.0	p=5.12e-11	*
2ms	low vs. low	12.24	11.78	-0.46	U=1082.5	p=0.234	n.s.
2ms	mod vs. mod	12.32	13.96	1.64	U=2237.0	p=2.51e-12	*
2ms	high vs. high	12.34	12.54	0.2	U=1356.0	p=0.433	n.s.

Supplementary Table S2: **Comparison of dimensionality at 10s- vs. 8h-recall for 2ms bins (related to Supplementary Figure S4a,b,c).** Table shows the results of Mann-Whitney U tests as well as relevant means, mean differences, and p-values. None, low, moderate, and high correspond to gray, yellow, green, and blue stars in Supplementary Figure S4a,b,c. Sample size is 50 network simulations per condition.

bin size	neuromodulator	mean 10s	mean 8h	mean 8h - mean 10s	mann whitney u	p-value	sig. at α = 0.001
4ms	none vs. none	3.74	1.54	-2.2	U=106.0	p=4.29e-16	*
4ms	low vs. low	3.58	2.26	-1.32	U=253.0	p=2.32e-13	*
4ms	mod vs. mod	3.52	4.14	0.62	U=1794.0	p=4.84e-05	*
4ms	high vs. high	3.62	3.34	-0.28	U=911.5	p=0.007	n.s.

Supplementary Table S3: **Comparison of dimensionality at 10s- vs. 8h-recall for 4ms bins (related to Supplementary Figure S4d,e,f).** Table shows the results of Mann-Whitney U tests as well as relevant means, mean differences, and p-values. None, low, moderate, and high correspond to gray, yellow, green, and blue stars in Supplementary Figure S4d,e,f. Sample size is 50 network simulations per condition.

bin size	neuromodulator	mean low	mean mod/high	mod/high - low	mann whitney u	p-value	sig. at α = 0.001
1ms	low vs. mod	13.2	21.16	7.96	U=0.0	p=4.53e-18	*
1ms	low vs. high	13.2	20.76	7.56	U=0.0	p=4.16e-18	*
2ms	low vs. mod	11.78	13.96	2.18	U=206.0	p=1.98e-13	*
2ms	low vs. high	11.78	12.54	0.76	U=802.0	p=0.001	*
4ms	low vs. mod	2.26	4.14	1.88	U=133.5	p=1.03e-15	*
4ms	low vs. high	2.26	3.34	1.08	U=285.0	p=5.64e-13	*

Supplementary Table S4: **Comparison of dimensionality for low vs. moderate and low vs. high neuromodulator levels at 8h-recall (related to Figure 5 in main article and Supplementary Figure S4).** Table shows the results of Mann-Whitney U tests as well as relevant means, mean differences, and p-values. None, low, moderate, and high correspond to gray, yellow, green, and blue stars in Figure 5 in main article for 1ms bins, Supplementary Figure S4a,b,c for 2ms bins, and Supplementary Figure S4d,e,f for 4ms bins. Sample size is 50 network simulations per condition.
